# Case report: Transfusion independence and abolition of extravascular hemolysis in a PNH patient treated with pegcetacoplan

**DOI:** 10.3389/fimmu.2022.1060923

**Published:** 2022-12-01

**Authors:** Bruno Fattizzo, Francesco Versino, Anna Zaninoni, Anna Paola Maria Luisa Marcello, Cristina Vercellati, Silvia Artuso, Wilma Barcellini

**Affiliations:** ^1^ Department of Oncology and Oncohematology, University of Milan, Milan, Italy; ^2^ Hematology Unit, Fondazione Istituto di Ricovero e Cura a Carattere Scientifico (IRCCS) Ca’ Granda Ospedale Maggiore Policlinico, Milan, Italy

**Keywords:** pegcetacoplan, paroxysmal nocturnal hemoglobinuria, extravascular hemolysis, direct antiglobulin test, ektacytometry

## Abstract

More than half of patients with paroxysmal nocturnal hemoglobinuria (PNH) treated with complement fraction C5 inhibitors experience residual anemia and hemolysis. This is partly due to the persistent activation of the complement cascade upstream C5, resulting in C3 deposition on PNH erythrocytes and extravascular hemolysis in the reticuloendothelial system. Pegcetacoplan is the first proximal C3 inhibitor to be approved for PNH basing on favorable efficacy and safety data in both naïve and eculizumab treated PNH. Here we report the first Italian patient treated with pegcetacoplan in a named patient program. The patient suffered from hemolytic PNH associated with CALR+ myeloproliferative neoplasm and was heavily transfusion dependent despite eculizumab therapy. Treatment with pegcetacoplan induced a dramatic improvement in Hb, along with normalization of unconjugated bilirubin and reticulocytes, as markers of extravascular hemolysis. Sequential laboratory workup showed the disappearance of C3 deposition on erythrocytes by direct anti-globulin test, the increase of PNH clone on erythrocytes, and a peculiar right shift of the ektacytometry curve. The drug was well tolerated, and the patient reported a significant improvement in his quality of life. Overall, pegcetacoplan appears a safe and effective option “ready to use” in the clinic for patients with PNH and suboptimal response to anti-C5 agents.

## Introduction

Pegcetacoplan is the first proximal inhibitor to be licensed for the treatment of paroxysmal nocturnal hemoglobinuria (PNH) in USA and Europe. It is a compstatin analog consisting of a 13-residue disulfide-bridged peptide, modified through pegylation, that binds to C3 and C3b, preventing C3 convertase activity and thus blocking the complement cascade (1). Pegcetacoplan is able to prevent C3 deposition on red blood cells (RBC) thus reducing extravascular hemolysis (EVH), one of the major causes of suboptimal response to anti-C5 monoclonal antibodies eculizumab and ravulizumab. Pegcetacoplan was effective in alleviating anemia and transfusion need in PNH patients already on treatment with eculizumab, as well as in those naïve to complement inhibitor in registration trials (2, 3); however, real-life evidence is still limited. Here we describe the outcome of the first Italian patient treated with pegcetacoplan in a named patient program. We confirmed the clinical efficacy of the drug in a complex scenario of transfusion dependent PNH associated with CALR mutated myeloproliferative neoplasm (MPN). Additionally, we explored drug effect on RBC morphologic and functional properties, including ekacytometry. A brief review of published data on pegcetacoplan use in PNH is also provided.

## Methods

Written informed consent was obtained from the individual for the publication of any potentially identifiable images or data included in this article. The investigation was conducted according to the Helsinki declaration and after approval of the local Ethical committee. All information regarding patient clinical history, hematologic parameters and bone marrow (BM) evaluation were prospectively collected from September 2020 until the time of writing. Direct anti-globulin test (DAT), complement fractions C3 and C4, red blood cell morphology on peripheral blood smear, and ektacytometric analysis were sequentially performed during pegcetacoplan therapy.

### Osmotic gradient ektacytometry (Osmoscan curve)

For ektacytometry, 250 μL of EDTA blood sample was suspended in 5 mL of polyvinylpyrrolidone buffer (PVP, Mechatronics, Hoorn, The Netherlands) and analysed by Laser-assisted Optical Rotation Cell Analyzer (LoRRca MaxSis, Mechatronics, Hoorn, The Netherlands) as previously described (4). The osmoscan curves reflect RBC deformability as a continuous function of suspending medium osmolality. The main parameters considered were Omin (which reflects the cellular osmotic fragility), the elongation index (EI) max (maximal deformability), and the Ohyper (which reflects mean cellular hydration status).

## Case description

**Figure 1:**
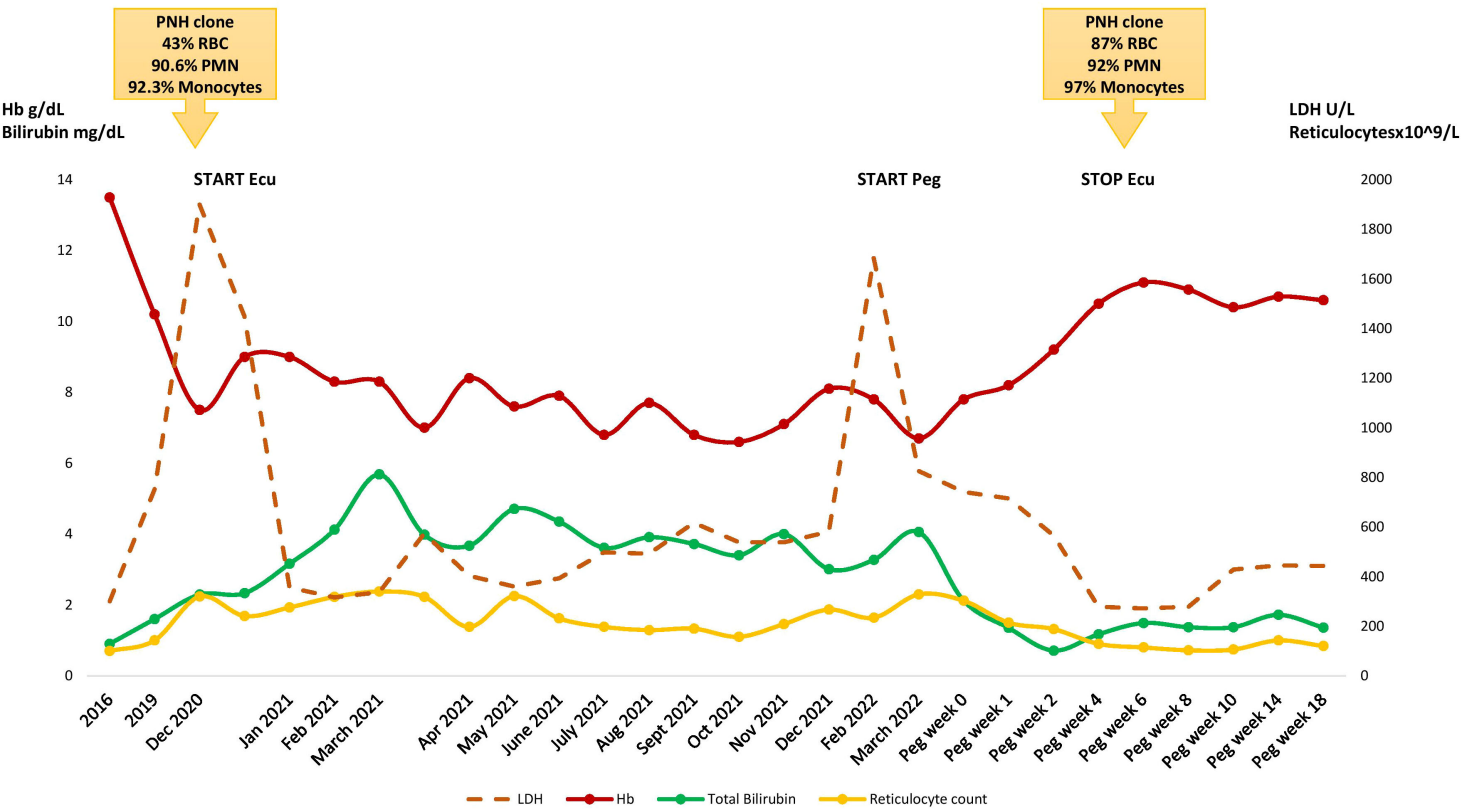
Hematologic parameters during the clinical course. RBC, red blood cells; PMN, polymorphonucleates; Peg, pegcetacoplan; Ecu, eculizumab.

A 79-year-old man was diagnosed in November 2020 with PNH. His past medical history included hypertension, prostate adenocarcinoma treated in 2017 with surgery, and recurrent basocellular carcinomas. In 2007 he had received a diagnosis of CALR mutated essential thrombocythemia (ET) and was put on hydroxyurea with good response. In 2016 an isolated moderate LDH increase (i.e. 1.6x ULN) occurred, and was later accompanied by mild unconjugated hyperbilirubinemia and progressive decrease of Hb values (**Figure 1**). Since October 2019 overt hemolysis was present along with appearance of transfusion need (1 RBC unit every 2 months). Both direct (DAT) and indirect antiglobulin tests were negative. Endogenous erythropoietin values were normal and no sign of organomegalies were found at the abdomen ultrasound. A new bone marrow trephine showed trilinear hypercellularity and increased marrow fibrosis, consistent with a fibrotic evolution of ET. JAK2 inhibitor was not proposed due to absence of splenomegaly and the presence of severe cytopenias. The patient received a trial with steroids 1mg/kg for hemolytic anemia without benefit at the local center, followed by danazol 400mg/day (the latter from February to July 2020), again with no response. Given the presence of steroid-resistant DAT-negative hemolytic anemia, in November 2020 FLAER analysis was done at our center and demonstrated the presence of a large PNH clone in 43% of RBC, 90.6% of granulocytes, and 92.3% of monocytes. After anti-meningococcal vaccination, in December the patient started eculizumab (900 mg iv fortnightly) due to transfusion dependent (1 RBC units/month) hemolytic PNH (LDH 1900 U/L, unconjugated bilirubin 6 mg/dL, increased reticulocytes and consumed haptoglobin). Notably, prophylactic anticoagulation therapy with low molecular weight heparin was administered from PNH diagnosis until eculizumab start and LDH normalization.

Eculizumab was only partially effective, LDH normalized (suggesting that LDH elevation was chiefly due to PNH and not to MPN), but anemia persisted, along with hyperbilirubinemia and consumed haptoglobin. Transfusion need progressively worsened to 2 RBC units/month. A further bone marrow biopsy and PNH clone size were unchanged; DAT re-evaluation detected C3 deposition consistent with C3-mediated extravascular hemolysis, and serum C3 levels were reduced (83 mg/dL, normal range 90-180 mg/dL). Due to concomitant MPN the patient was not eligible for clinical trials. Therefore, an expanded access program for pegcetacoplan was activated. In March 2022 the patient received anti-Haemophilus and anti-pneumococcal vaccines and started pegcetacoplan as add on to eculizumab. Hemolysis initially worsened due to intercurrent fever of unknown origin (i.e. pharmacodynamic breakthrough hemolysis, BTH), but thereafter Hb improved and the patient became transfusion independent soon after the second pegcetacoplan dose (7 days). After less than one month of treatment the patient displayed stable Hb values >10g/dL, normal hemolytic markers and C3 levels. Eculizumab was stopped in April 2022, and the patient continued subcutaneous pegcetacoplan 1,080 mg twice a week, with sustained hematological response and without adverse events (last follow up September 2022 Hb 10.6 g/dL and normal hemolytic markers). Finally, the PNH clone size on RBCs increased from 43% to 87% after pegcetacoplan.

**Figure 2:**
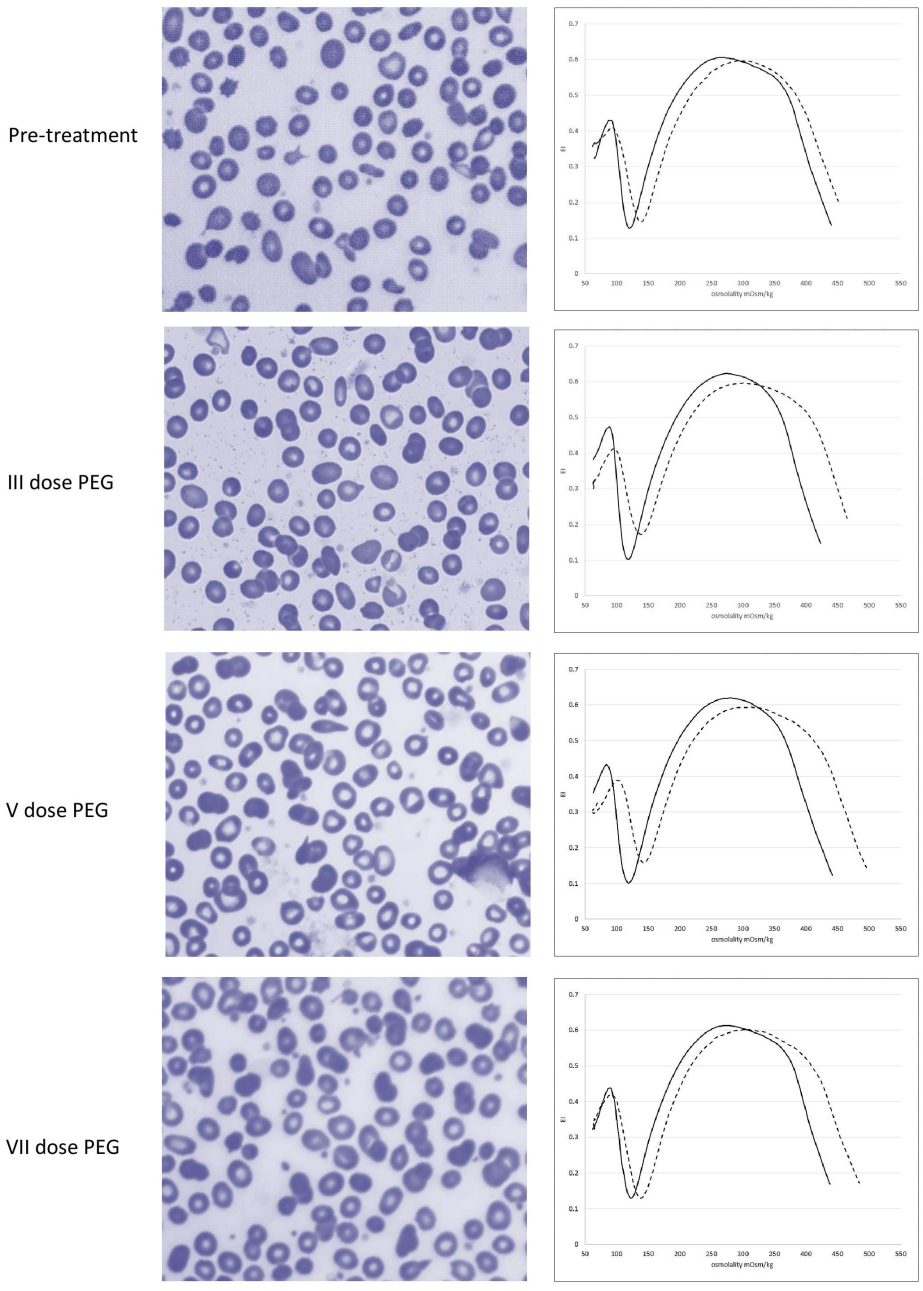
Erythrocyte morphology and ektacytometry before and after pegcetacoplan treatment.

Serial DAT evaluations at 1, 3, and 6 weeks after pegcetacoplan start, confirmed negativity of C3 deposition on patient’s RBCs. **Figure 2** shows sequential peripheral blood smear morphology with the persistence of ovalocytes, spherocytes, dacrocytes, and stomatocytes before and after treatment, consistent with the underlying diagnosis of MPN. Notably, pre-treatment osmoscan curve showed a slight right shift, as observed in cases of overhydrated RBC; after the 1^st^ and subsequent doses of pegcetacoplan, the right shift further increased (Ohyper values from 431 mOsm/kg pre-treatment to 450, 463, and 453, at the 3^nd^, 5^th^, and 7^th^ dose, respectively, **Figure 2**). This may be due to the increased size of RBC PNH clone while on treatment, resulting in an osmoscan curve with Ohyper being significantly different compared to controls as already reported for PNH (4).

## Review of the literature

**Table 1** summarizes the available evidence on pegcetacoplan use in PNH. The drug was firstly investigated in two phase I trials in healthy volunteers (5) and showed a good safety profile both as single and multiple ascending doses. Subsequently, the PADDOCK (NCT02588833) and PALOMINO (NCT03593200) phase I/II trials evaluated untreated PNH patients and demonstrated a normalization of hemoglobin levels with consequent transfusion independency. Furthermore, normalization of extravascular hemolytic markers, and the increase of proportion of PNH erythrocytes, were consistent with the ability of pegcetacoplan to control both intravascular hemolysis and EVH. Pegcetacoplan appeared generally safe, although drug-related hypersensitivity events occurred in the PADDOCK study. Importantly, there were no thrombotic events or severe infectious complications (6). Another phase Ib study, the PHAROAH trial (NCT02264639), investigated pegcetacoplan as an add-on therapy in suboptimal responders to eculizumab and demonstrated a hematological improvement, enabling eculizumab discontinuation in 4 subjects (7). Based on these studies, two phase III trials were designed: the PRINCE (NCT04085601) study compared pegcetacoplan to standard of care in naïve PNH subjects, showing good tolerability and efficacy (2). The PEGASUS (NCT03500549) trial enrolled suboptimal responders to eculizumab (Hb<10.5 g/dL): during the first 4 weeks, patients received pegcetacoplan 1,080 mg biweekly in addition to eculizumab. Thereafter, they were randomized to receive either pegcetacoplan or eculizumab for 16 weeks. The study demonstrated that pegcetacoplan contributed to superior improvements in Hb levels and transfusion independency compared to eculizumab, without major treatment emergent adverse events. Results from the 32- and 48-weeks follow-up confirmed the long-term safety and durable efficacy of pegcetacoplan (3). Based on these data, pegcetacoplan was approved by FDA in May 2021 for adults with PNH, and by EMA in December 2021 for those remaining anemic after at least 3 months of stable C5 inhibitors. In the absence of a head-to-head comparison, Bhak RY et al. compared pegcetacoplan versus ravulizumab by reanalyzing phase 3 results with matching-adjusted indirect comparison methodology, and confirmed the superiority of the former (8). A case report of one young PNH patient with C5 genetic variant who benefited from pegcetacoplan therapy was also published (9). Finally, the UK experience on pegcetacoplan compassionate use was recently published confirming safety and efficacy of the drug in this patient population (10).

**Table 1:** Literature review regarding pegcetacoplan use in paroxysmal nocturnal hemoglobinuria.

Reference	Trial name	N° of patients	Patient population	Phase of study	Comments
Grossi FV et al., Blood 2016 (5)	–	51	Healthy volunteers	Phase I	Single and multiple doses of Peg were safe; serum concentrations close to steady state reached after 28 days of daily dosing. A reduction in AP50 was seen following a single dose of 1440mg and multiple daily doses of 180 and 270 mg.
Wong RSM, et al, Annals of Hematology 2022 (6)	PADDOCK	26	PNH naive	Phase Ib	Hemolytic markers and Hb values normalization with Peg, 65% achieved transfusion independence. One hypersensitivity event related to Peg. No thrombotic events or infectious complications.
Wong RSM, et al, Annals of Hematology 2022 (6)	PALOMINO	4	PNH naive	Phase IIa	100% transfusion independence. No SAE related to Pegcetacoplan.
De Castro C et al, American J. of Hematology 2020 (7)	PHAROAH	6	PNH on C5-inhibitor	Phase Ib	Pegcetacoplan improved hematological response and enabled eculizumab discontinuation and transfusion independence in 4 patients.
Wong RSM et al, Ther Adv Hematology 2022 (2)	PRINCE	53	PNH naive	Phase III	Pegcetacoplan vs SoC excluding complement inhibitors. Hb and LDH normalization in 45.7% and 65.7% of patients respectively with Peg, 0% with SoC. No SAE registered.
Hillmen P et al, NEJM 2021 (3)	PEGASUS	80	PNH on C5-inhibitor	Phase III	Pegcetacoplan proved superior to eculizumab in Hb increase and transfusion independence. Safety profile comparable to eculizumab, and lower rate of BTH with pegcetacoplan. Overall, in patients with suboptimal response to eculizumab, pegcetacoplan treatment is effective and well tolerated.
Bhak RY et al, Blood 2022 (8)	PEGASUS and 302	69 and 195	PNH on C5-inhibitor	Matching-adjusted indirect comparison	MAIC allowed examination of the comparative effectiveness of pegcetacoplan vs ravulizumab in the absence of a head-to-head trial. Pegcetacoplan superior to ravulizumab in terms of transfusion avoidance.
Tamura S et al, British J. of Hematology 2022 (9)	–	1	PNH on C5-inhibitor	Case Report	Pegcetacoplan effective in one patient with PNH and C5 genetic variant, non-responding to eculizumab.

Peg, pegcetacoplan; PNH, paroxysmal nocturnal hemoglobinuria; SoC, standard of care; SAE, serious adverse events; MAIC, matching adjusted indirect comparison; BTH, breakthrough hemolysis.

## Discussion and conclusions

Here we confirm the beneficial effect of pegcetacoplan in a complex setting of PNH associated with MPN, and suboptimal response to standard anti-C5 therapy. Suboptimal response to eculizumab in this patient may have reckoned several causes including residual terminal C5 activation, the concomitant myeloid neoplasm, and C3 mediated EVH. The latter was evidenced as the prevailing mechanism, by multiple systematic re-evaluations of hemolytic markers, bone marrow trephine, DAT and C3 and C4 levels. Consistently, a complete normalization of bilirubin, reticulocytes and haptoglobin, DAT negativity, complement levels recovery, and increase of RBC PNH clone size by flow cytometry and by ektacytometry analysis were observed. Importantly, the patient obtained transfusion independence and Hb levels even higher than those before eculizumab. Finally, the amelioration of quality of life emerging throughout the published studies was confirmed in our elderly patient. In fact, despite the concerns regarding the need for biweekly subcutaneous injections, no difficulties were reported in self-administration and improved convenience was noted.

Regarding the association with the underlying myeloproliferative syndrome, we did not evaluate the clonal relationship between PIG-A and CALR mutated hematopoietic stem cells. Heterogeneous mutational patterns have been described for PNH and bone marrow failures, with PIGA mutation possibly coexisting, preceding, or following the myeloid gene lesions (11). Notably, no progression of the myeloid neoplasm has been observed during anti-complement therapy so far.

Regarding safety, the febrile episode associated with BTH observed after the first administration of the drug deserves some consideration. Literature review does not report an increased infectious risk with pegcetacoplan even in association with anti-C5, and provided triple anti-capsulated vaccinations not mandatory with anti-C5 only (9). However, the limited follow up requires confirmatory data from real world studies. Concerning BTH, it has been reported in pegcetacoplan clinical trials and has been also related to the short half-life of the drug (2, 3). Again, there are no clear recommendations on the management of BTH in this setting (additional dose of anti-C3? rechallenge with anti-C5?). A final open issue is the prevention of thrombosis with pegcetacoplan in the long term (12). Nevertheless, pegcetacoplan appears a safe and effective option “ready to use” in the clinic, awaiting novel oral and subcutaneous agents.

## Data availability statement

The raw data supporting the conclusions of this article will be made available by the authors, without undue reservation.

## Ethics statement

The studies involving human participants were reviewed and approved by Comitato Etico Milano Area 2. The patients/participants provided their written informed consent to participate in this study. Written informed consent was obtained from the individual for the publication of any potentially identifiable images or data included in this article.

## Author contributions

BF, VF, and WB followed the patient and wrote the article. AZ, AM, and CV performed erythrocyte morphology and ektacytometry and wrote the manuscript. SA registered the patient in the named patient program and wrote the article. All Authors revised the manuscript for important intellectual content and approved the final version.

## Funding

The manuscript was partially funded by the Italian Ministry of Health, Current Research 2021.

## Conflict of interest

BF received consultancy from Apellis, Momenta, Novartis, Amgen, Sobi and Janssen and lecture fee/congress support from Alexion and Apellis. WB received consultancy from Agios, Alexion, Apellis, Biocryst, Bioverativ, Incyte, Momenta, and Novartis; and lecture fee/congress support from Alexion, Incyte, Novartis, and Sanofi.

The remaining authors declare that the research was conducted in the absence of any commercial or financial relationships that could be construed as a potential conflict of interest.

## Publisher’s note

All claims expressed in this article are solely those of the authors and do not necessarily represent those of their affiliated organizations, or those of the publisher, the editors and the reviewers. Any product that may be evaluated in this article, or claim that may be made by its manufacturer, is not guaranteed or endorsed by the publisher.
